# Feasibility of Serum and Faecal Interleukin Assessment in Equine Surgical Colic: A Pilot Study

**DOI:** 10.3390/ani16213396

**Published:** 2026-10-28

**Authors:** Erwan Degorce, Daria Lorek, Agnieszka Żak-Bochenek, Dorota Długopolska, Paweł Golonka, Artur Niedźwiedź, Natalia Siwińska

**Affiliations:** 1 Faculty of Veterinary Medicine, Wroclaw University of Environmental and Life Sciences, 50-375 Wroclaw, Poland; 2 Department of Immunology, Pathophysiology and Veterinary Preventive Medicine, Wroclaw University of Environmental and Life Sciences, 50-375 Wroclaw, Poland; daria.lorek@upwr.edu.pl (D.L.);; 3 Department of Internal Medicine and Clinic of Diseases of Horses, Dogs and Cats, Faculty of Veterinary Medicine, Wroclaw University of Environmental and Life Sciences, 50-366 Wroclaw, Polandartur.niedzwiedz@upwr.edu.pl (A.N.); 4 Equivet Szpital dla Koni, 55-216 Gesice, Poland

**Keywords:** equine colic, cytokines, faecal biomarkers, sampling feasibility, interleukins, gastrointestinal inflammation

## Abstract

Cytokines may provide information about inflammation in equine colic, but their clinical relevance remains uncertain. This pilot study assessed short-term changes in serum and faecal interleukin (IL)-1β, IL-6 and IL-8 in eight horses undergoing colic surgery and compared measurements with six healthy controls. Colic horses were sampled at admission (D0), 24 h (D1) and day 7 (D7); controls provided serum at D0 and faeces at all three time points. Cytokines were assayed using MILLIPLEX and MAGPIX. Admission faeces were unavailable from 3/8 colic horses (37.5%). Serum IL-1β and IL-6 were below detection limits in 29/29 analysed samples (100%) for each cytokine. Serum IL-8 varied markedly without significant temporal change. Thirty-nine faecal extracts were analysed without faecal assay validation. Faecal estimates were generally lower in colic horses, with nominal between-group differences for IL-6 at D1 (*p* = 0.045), IL-1β at D7 (*p* = 0.033) and IL-8 at D7 (*p* = 0.004). No consistent temporal pattern in faecal estimates or significant association between serum and faecal IL-8 was detected. These findings do not establish the usefulness of these cytokines for clinical monitoring and highlight sampling and analytical limitations that should be addressed in larger, clinically diverse cohorts.

## Simple Summary

Monitoring inflammation could help veterinarians assess recovery in horses undergoing surgery for colic. This pilot study examined three immune signalling molecules, interleukin (IL)-1β, IL-6 and IL-8, in blood serum and faeces from eight horses undergoing colic surgery and six healthy horses. Measurements at admission, 24 h and seven days were compared. Serum IL-1β and IL-6 were below the test’s detection limits in all analysed samples, while serum IL-8 varied widely. Faecal measurements were generally lower in horses with colic and showed no consistent pattern over time. However, the test’s accuracy for measuring these molecules in horse faeces had not been established, so the results require caution. Faeces could not be collected from some horses at admission, and all horses with colic had received treatment before sampling. The study therefore does not show that these measurements can reliably monitor recovery after colic surgery. It highlights practical and laboratory limitations that should be addressed in larger studies including horses with varying disease severity.

## 1. Introduction

Colic is a major cause of emergency presentation and mortality in horses [1,2]. Gastrointestinal distension, impaction, displacement and strangulation can cause local tissue injury and inflammation and, in severe cases, endotoxaemia and systemic inflammatory response syndrome [3,4,5]. Clinical examination, rectal palpation, ultrasonography and routine laboratory tests remain central to assessment, while biomarkers such as lactate and acute-phase proteins can provide complementary information [6,7]. Additional markers that are reproducible, rapidly measurable and clinically accessible could improve characterisation and monitoring of acute abdominal disease.

Interleukin (IL)-1β, IL-6, and IL-8 are inflammatory mediators with complementary functions: IL-1β contributes to the initiation and amplification of inflammation, IL-6 mediates acute-phase responses, and IL-8 promotes neutrophil recruitment and activation [8,9,10,11,12,13]. These functions, together with previous equine findings, provided the rationale for selecting this focused cytokine panel. Increased expression of all three cytokine genes has been reported during the early perioperative period in 15 horses undergoing colic surgery [14], while increased blood and peritoneal fluid IL-6 activity has been documented in a study of 155 horses with acute abdominal disease [15]. However, gene expression and biological activity are not directly interchangeable with protein concentrations, and cytokine measurements may vary with lesion type, sample material, assay, and sampling time [15,16]. Human studies demonstrating faecal cytokines, including increased stool IL-1β in active inflammatory bowel disease [17,18], provide additional rationale for exploring faeces as a potentially non-invasive source of inflammatory markers. These findings do not validate faecal cytokine measurements in horses, for which data on IL-1β, IL-6, and IL-8 and their temporal changes in surgical colic remain limited.

This pilot study aimed to evaluate short-term changes in serum and faecal IL-1β, IL-6, and IL-8 concentrations in horses undergoing surgery for colic and to compare these concentrations with those in healthy horses. It was hypothesised that serum and faecal interleukin concentrations would be higher in horses with colic than in healthy horses and would be associated with clinical status. Concentrations were expected to be elevated before surgery and during the early postoperative period, subsequently declining as inflammation subsided and approaching levels observed in healthy horses. Such a pattern could support the potential use of these cytokines as indicators of clinical status and postoperative recovery in horses with surgical colic. Faeces could potentially provide a non-invasively collected sample for assessing the degree of inflammation in these horses. The study was exploratory and was not designed to establish prognostic thresholds or associations with lesion severity.

## 2. Materials and Methods

### 2.1. Horses

The study included eight horses with acute colic requiring surgical intervention and six healthy control horses presented to Equivet Szpital dla Koni (Poland) for a general health check-up, orthopaedic physiotherapy, or rehabilitation. Individual horses were assigned unique identifiers prefixed with C (colic) or H (healthy controls), which are used throughout the manuscript. Controls had no gastrointestinal signs and had received no systemic medication, including antibiotics, anti-inflammatory or antiulcer drugs, or probiotics, during the preceding six months. Age, sex and available breed information were recorded for both groups. For colic horses, clinical examination, rectal and abdominal ultrasonographic findings, laboratory data, treatment, surgical diagnosis/procedure and postoperative course were recorded. Horses with colic were referred to the clinic with a preliminary diagnosis and an identified need for surgical intervention. All had received analgesic and anti-inflammatory medication and fluid therapy before the first blood sample was collected. The interval from admission to surgery was typically 30–60 min and included clinical assessment, diagnostic investigations, drug administration, and preparation for surgery. Clinical treatment during hospitalisation was individualised according to lesion type, disease severity, and patient requirements, rather than standardised for this study. Analgesic and anti-inflammatory treatment included flunixin (administered as flunixin meglumine; 1.1 mg/kg IV; Vetaflunix 50 mg/mL VET-AGRO, Lublin, Poland) and/or metamizole sodium monohydrate (20–50 mg/kg IV; Biovetalgin 500 mg/mL Biowet Drwalew, Poland), with repeated administration according to clinical requirements. All horses in the surgical colic group received intravenous fluid therapy before and during surgery using crystalloid solutions (Optilyte, Ringer’s solution and/or 0.9% sodium chloride, Fresenius Kabi, Kutno, Poland). Fluid requirements were calculated individually for each horse based on maintenance needs, with additional allowances for estimated dehydration deficits and ongoing fluid losses. All horses, including the control horses, remained at the clinic throughout the seven-day study period and were discharged after the planned observation period ended or after required clinical treatment. In horses with colic, basic blood parameters, including total protein (TP), haematocrit (HT) and white blood cell count (WBC), were additionally assessed at admission (D0). The analysed cohort comprised horses followed through D7; horses that died before serial sampling could be completed were excluded.

In accordance with prevailing regulations, Ethics Committee approval was not required for this study. All samples were collected as part of routine veterinary procedures and did not cause pain, suffering, or stress equal to or greater than a needle puncture. Written consent was obtained from the horse owners for all sample collections.

### 2.2. Material Collection

**Figure 1:**
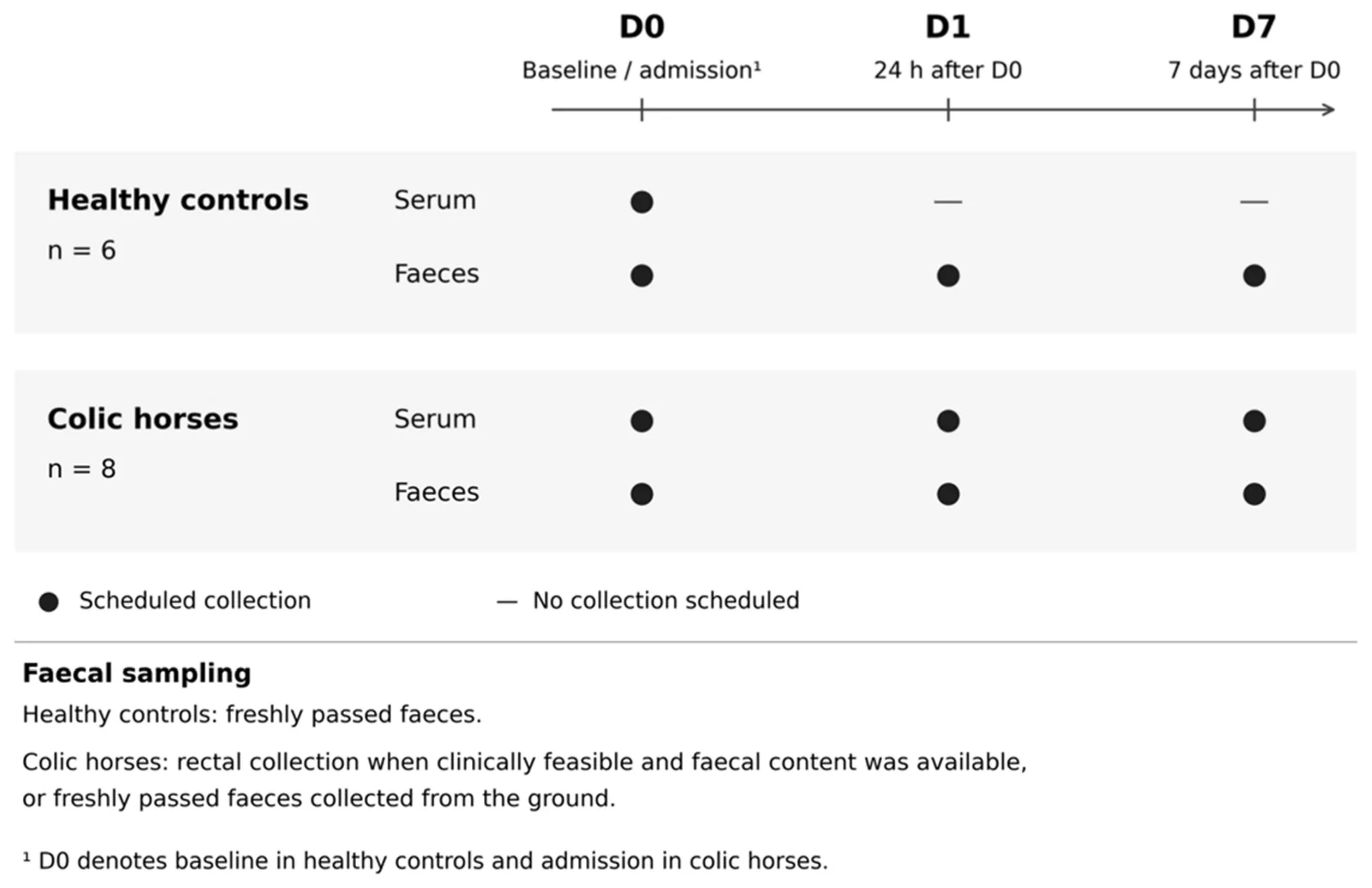
Planned sampling schedule for healthy control horses (*n* = 6) and horses with colic (*n* = 8). Not all samples could be obtained at each scheduled time point; the actual numbers of samples collected are reported in Section 3.1.

The planned sampling schedule for healthy control horses and horses with colic is presented in Figure 1. Blood samples were collected from control horses at D0 and from colic horses at admission (D0), 24 h later (D1), and on day 7 (D7), using a Vacutainer^®^ (Becton, Dickinson and Company, Franklin Lakes, NJ, USA) evacuated blood collection system with clot activator tubes. Blood was allowed to clot for at least 30 min and then centrifuged at 1000× *g* for 10 min to obtain serum. The separated serum was stored at −20 °C for 12 h and subsequently transferred, without thawing, to a −80 °C freezer until analysis.

Faecal samples were collected from both control and colic horses at D0, D1 and D7 when available. In control horses, samples were collected from freshly passed faeces. In colic horses, samples were obtained rectally when clinically feasible and faecal content was available, or from freshly passed faeces collected from the ground. Thus, faecal sampling at D0 depended on faecal availability and the safety of collection. Five grams of fresh faeces were mixed with 5 mL of phosphate-buffered saline (PBS) containing 1% protease inhibitor cocktail (539131, EMD Millipore Corp., Darmstadt, Germany), shaken and centrifuged at 1600× *g* for 20 min. The supernatant was filtered through a 0.45 μm filter. Filtered faecal supernatants underwent the same storage procedure as serum: 12 h at −20 °C, followed by transfer without thawing to −80 °C until analysis. Serum and faecal supernatants were thawed only once, immediately before laboratory analysis.

### 2.3. Cytokine Analysis

Serum and faecal IL-1β, IL-6, and IL-8 were measured in duplicate using the MILLIPLEX MAP Equine Cytokine/Chemokine Magnetic Bead Panel (EQCYTMAG93K; Merck KGaA, Darmstadt, Germany) and a Luminex^®^ MAGPIX^®^ analyser (Luminex Corporation, Austin, TX, USA). Samples (25 μL) were run neat with overnight incubation. A minimum bead count of 50 per cytokine and a duplicate coefficient of variation <10% were required for inclusion. Belysa^®^ Software (Merck KGaA, Darmstadt, Germany) was used to process multiplex assay output and derive concentration estimates from the assay calibration curves. The assay was not specifically validated for equine faecal extracts: recovery, dilutional linearity and potential interference from components of the faecal extracts were not assessed, and limits of detection and quantification specific to these extracts were not established. Faecal results were therefore interpreted as exploratory assay-derived estimates, with caution regarding their quantitative accuracy.

### 2.4. Statistical Analysis

Normality was assessed using the Shapiro–Wilk test. Serum IL-8 concentrations at D0 were compared between control and colic horses using Student’s *t*-test. Faecal cytokine concentrations were compared between groups at individual time points using the Mann–Whitney U test. Serial changes within groups were assessed using Friedman ANOVA, and serum–faeces IL-8 associations were evaluated using Spearman’s rank correlation. Concentrations are reported as median and interquartile range (Q1–Q3). Associations between D0 faecal cytokine concentrations or serum IL-8 and concurrent HT, TP and WBC in horses with colic were assessed using Spearman’s rank correlation with available complete pairs. These analyses were performed using exact two-sided permutation tests, with Holm adjustment across the 12 comparisons. Because of the small pilot cohort and the number of exploratory comparisons, *p* values were interpreted as nominal rather than confirmatory. Analyses were performed in Statistica, with *p* < 0.05 used as the nominal significance threshold. Serum IL-1β and IL-6 were not subjected to concentration-based statistical comparisons because all results were below the detection limits. No numerical values were substituted for below-detection results or missing measurements, and no imputation was performed.

## 3. Results

### 3.1. Horses

The control group comprised two females and four males, and the colic group comprised four females and four males. The mean age was 8.0 years (range 6–10 years) in the control group and 9.0 years (range 3–15 years) in the colic group. All horses included in the study were warmblood-type horses. All control horses were Polish sport horses (warmblood crossbreds). C2, C8 and C13 were described as Polish sport horses (warmblood crossbreds), C7 as Oldenburg, and C9, C10 and C12 as Polish noble half-bred horses. The specific breed of C16 was not established. Clinical diagnoses, procedures and postoperative courses are summarised in Table 1. No intestinal resection was performed in any of the eight included colic horses. C8 showed transient deterioration at 24 h followed by recovery; C2 and C12 developed postoperative complications, and C16 experienced mild postoperative pain. All eight colic horses were followed through D7.

At D0, faecal samples could not be obtained from C2, C8 or C9 (3/8; 37.5%). In C8 and C9, faecal material was absent from the rectum, whereas in C2, rectal examination could not be performed safely. The lack of these samples was therefore directly related to the acute gastrointestinal condition rather than to laboratory processing. Faecal samples were available from all colic horses at D1 and D7. Serum samples were collected from all colic horses at D0, D1 and D7; however, the sample from C9 at D1 was damaged and could not be analysed.

At admission (D0), the eight horses with colic had median (Q1–Q3) TP, HT and WBC values of 6.90 (6.50–7.10) g/dL, 35.00 (32.00–37.25) % and 10.55 (8.86–12.64) ×10^9^/L, respectively.

### 3.2. Interleukin Concentration Results

#### 3.2.1. Serum

**Figure 2:**
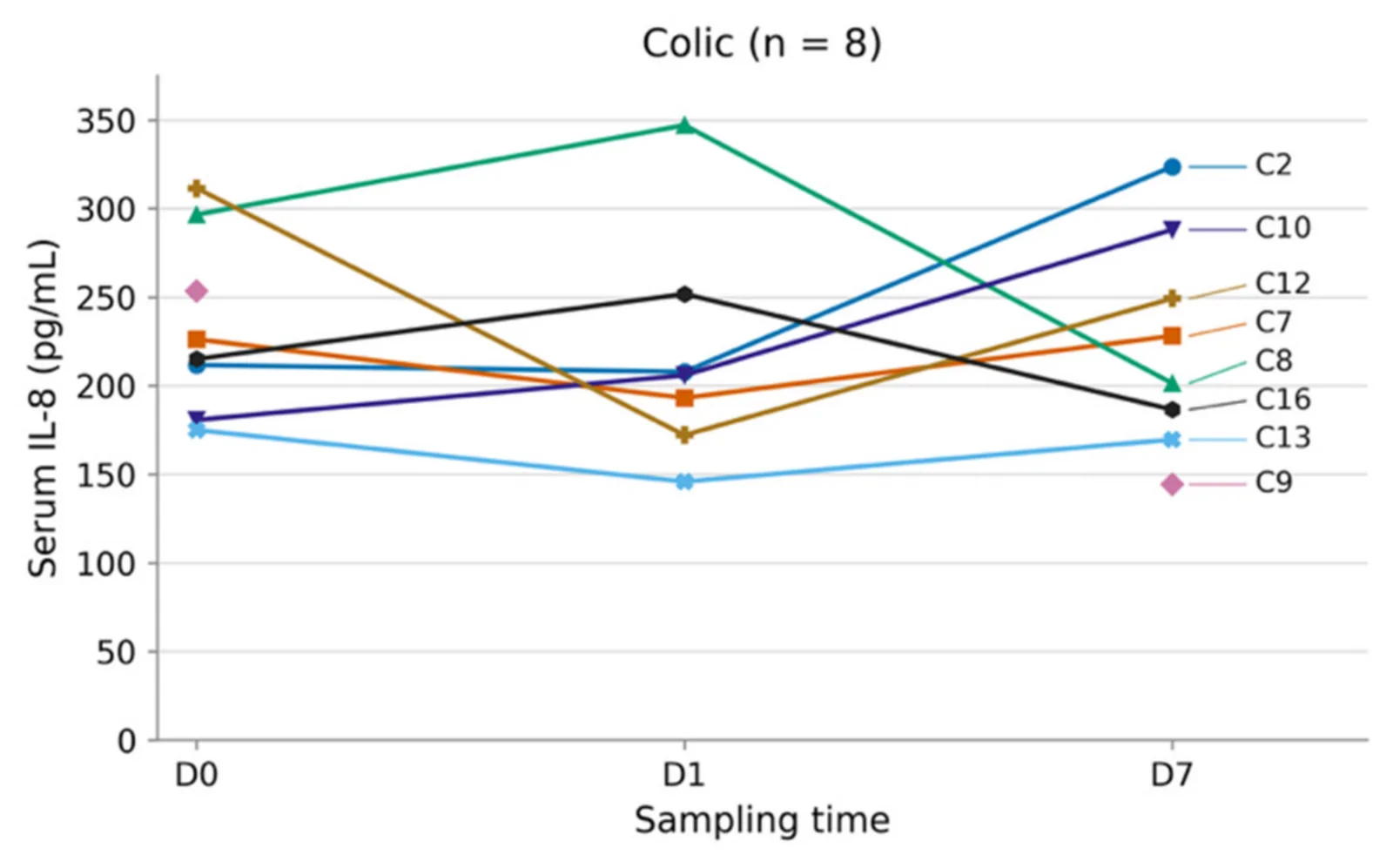
Individual trajectories of changes in IL-8 concentration in the serum of colic horses at time points D0, D1 and D7.

Serum IL-1β and IL-6 concentrations were below the respective assay detection limits in 29/29 analysed serum samples (100%) for each cytokine; neither was detectable (0/29, 0%). A total of 29 serum IL-8 results were obtained: six from healthy controls (6/6) and 23 from horses with colic (23/24), with the damaged C9 D1 sample excluded as described in Section 3.1. Serum IL-8 values showed marked inter-individual variability (Table 2 and Figure 2). The D0 difference between control and colic horses was not significant (*p* = 0.165), and no significant temporal change was identified in the colic group between D0, D1 and D7 (Friedman χ^2^ = 0.857, *p* = 0.651; Kendall’s W = 0.061).

#### 3.2.2. Faeces

**Figure 3:**
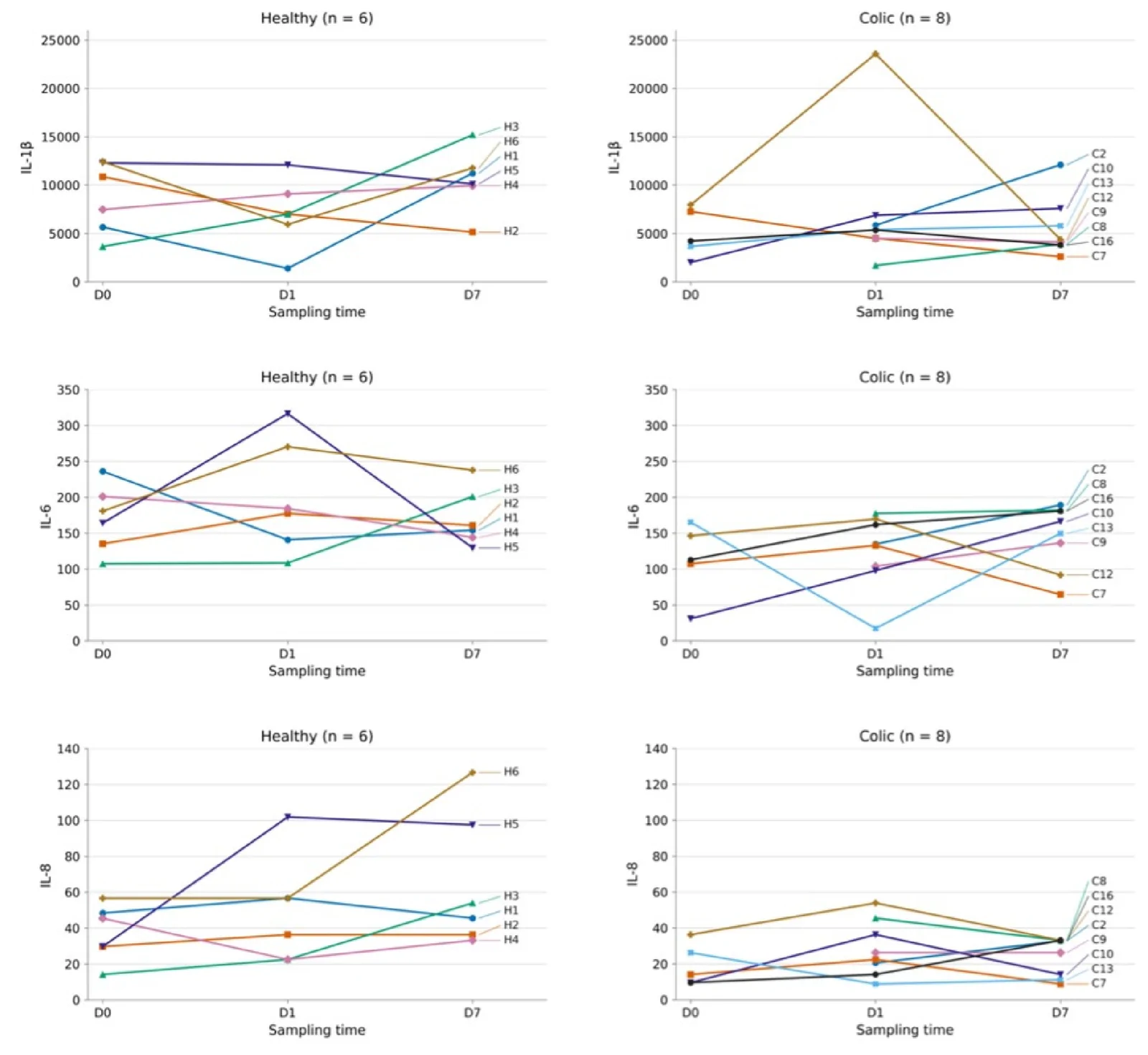
Individual trajectories of changes in IL-1β, IL-6, and IL-8 faecal estimates (pg/mL of extract) in healthy control horses (*n* = 6; **left panels**) and horses with colic (*n* = 8; **right panels**).

Faecal IL-1β, IL-6, and IL-8 were assayed in 39 available faecal extracts (control 18/18, colic 21/24 planned samples). These totals represent repeated samples from six control and eight colic horses, not independent horses. Results are summarised in Table 3 and Figure 3.

Faecal cytokine estimates showed marked inter-individual variability. Median values were lower in colic horses than in healthy controls for most cytokines and time points, but between-group differences reached nominal significance only for IL-6 at D1 (*p* = 0.045), IL-1β at D7 (*p* = 0.033) and IL-8 at D7 (*p* = 0.004). No significant temporal changes in faecal IL-1β, IL-6 or IL-8 were detected within either group (all *p* > 0.05), and no consistent unidirectional pattern was evident across horses.

#### 3.2.3. Serum–Faeces IL-8 Correlation

No significant correlation between serum and faecal IL-8 was detected at D0 (Spearman ρ = 0.359, *p* = 0.567), D1 (ρ = 0.143, *p* = 0.783) or D7 (ρ = 0.073, *p* = 0.887).

#### 3.2.4. Associations Between IL Concentrations and Basic Blood Parameters at D0

At D0, none of the associations between cytokine concentrations and HT, TP or WBC remained statistically significant after Holm adjustment. Faecal IL-6 was positively correlated with TP before adjustment (ρ = 0.975, n = 5, unadjusted *p* = 0.0333), but this association did not remain significant after correction (adjusted *p* = 0.400).

## 4. Discussion

This pilot study assessed short-term serum and faecal cytokine profiles in horses undergoing surgery for colic. The hypothesised elevation relative to healthy controls, followed by a return to comparable levels by D7, was not demonstrated. Serum IL-1β and IL-6 were below detection limits, while serum IL-8 and faecal estimates showed no consistent temporal pattern. Isolated nominal between-group differences in faecal values require cautious interpretation in view of the analytical limitations.

An important practical limitation was the inability to obtain faecal material at admission from three of eight horses with colic (37.5%). Sample availability depended on the gastrointestinal condition and the safety of collection. Intestinal motility and transit were not measured, and the absence of rectal faeces alone cannot establish the mechanism or severity of gastrointestinal dysfunction. Faecal biomarkers may preferentially be measurable in horses that are passing faeces or have rectal contents accessible for safe sampling. Horses without obtainable faeces may consequently be under-represented in admission comparisons, potentially introducing selection bias. This proportion should not be generalised to the wider colic population but highlights a clinically relevant sampling constraint.

The faecal cytokine estimates were also difficult to interpret biologically. Values were generally not increased in colic horses and varied markedly among individuals. Higher estimates in healthy controls should therefore not be interpreted as evidence of greater pathological intestinal inflammation. Faecal material collected distally may not represent an inflamed segment located more proximally, particularly when transit is impaired, and may reflect luminal conditions preceding the acute episode rather than inflammation at the time of sampling. Altered luminal flow and cytokine degradation could further weaken the relationship between local intestinal inflammation and the values measured in faeces. Differences in faecal water content could also influence the comparisons: extracting equal wet masses does not standardise dry matter content, and wetter samples could yield lower concentrations per volume of extract without indicating lower cytokine production. Differences in diet, microbiota and local immune regulation are additional possible contributors to baseline variability. The diverse biological roles of these cytokines also warrant caution when interpreting their presence as evidence of pathological inflammation [19,20,21]. These factors offer possible explanations for higher values in controls, but their contributions and direction of effect were not assessed in this study. As described in Section 2.3, the assay was not validated for equine faecal extracts. Differences in extraction recovery or assay interference therefore cannot be excluded as contributors to the observed variation. Enterotomy was performed in C8 and C16, but an association between these procedures and individual cytokine estimates was not evaluated. The observed group differences should therefore be regarded as exploratory rather than as direct measures of the relative severity of intestinal inflammation.

For serum measurements, differences among published equine studies may reflect assay platform, serum versus plasma, disease phenotype, and timing [15,22,23,24,25]. Postoperative IL-6 has been linked with complications after major abdominal surgery in humans [26], while Luminex-based equine plasma IL-8 measurements in other clinical populations further illustrate the importance of biological material and clinical context [27]. Cytokine responses can be brief: experimental endotoxaemia produces rapid changes in IL-1β, IL-6, and IL-8 gene expression [28], and IL-8 transcription after colic surgery can peak within hours [14]. Although transcriptional changes do not directly establish protein kinetics, sampling at admission, 24 h, and D7 may have missed an early systemic peak. The present findings should not be interpreted as evidence that these cytokines are uninvolved in colic pathophysiology; rather, their clinical utility could not be established in the sample types and time windows. At admission, cytokine concentrations were not significantly associated with HT, TP, or WBC after adjustment for multiple comparisons.

Clinical heterogeneity and treatment are additional sources of variability. The included horses had small- and large-intestinal lesions, and differences in lesion location, duration, and strangulation status may influence cytokine responses [15]. No intestinal resection was performed; however, possible strangulation in C7 could not be confirmed, and the cohort should not be classified as exclusively non-strangulating. None of the eight included horses was considered critically ill or showed clinical signs of endotoxaemia or marked haemodynamic compromise, limiting extrapolation to horses with more severe systemic involvement. Admission haematocrit values (median 35%; range 31–43%) did not suggest marked haemoconcentration, although haematocrit alone cannot exclude impaired perfusion or systemic inflammation. Pre-referral and subsequent hospital treatment may have modified cytokine responses [29,30,31,32]. Because all colic horses received medication and fluids before D0 sampling, admission measurements did not represent an untreated baseline. Crystalloid therapy may additionally have influenced serum concentrations through haemodilution. Treatment was individualised, and its effects could not be quantified or considered established explanations for the undetectable serum IL-1β and IL-6 results.

From a clinical perspective, sample preparation, particularly for faecal extracts, together with overnight incubation and the need for specialised laboratory equipment, limits the suitability of this approach for immediate decision-making in acute colic. Multiplex assay costs may also restrict routine use. Although turnaround time and costs were not formally evaluated in this study, both should be considered when assessing the potential value of these measurements for postoperative monitoring.

The small exploratory cohort limits statistical power, precision and subgroup assessment; non-significant findings do not demonstrate the absence of an association. Between 1 October and 31 March, 19 horses presenting with colic were admitted to the clinic and initially enrolled in the study. Three horses died and two were euthanised, resulting in an overall non-survival rate of 26.3% (5/19). Some horses were managed without surgery, others were discharged early, and in some cases sufficient serial samples could not be obtained according to the study schedule for various reasons. These factors reduced the final analysed cohort to eight surgically treated horses followed through D7, with the individual missing samples described above. Exclusion related to survival, discharge timing and sample availability introduces potential selection bias and limits generalisability to the wider surgical colic population, particularly severe or fatal cases. Blood and peritoneal lactate measurements were incomplete and were not included in the analysis, limiting assessment of cytokines in relation to perfusion and metabolic status. Lactate could provide complementary information on hypoperfusion and metabolic stress. In addition to the sampling, treatment, and faecal assay limitations discussed above, cytokine stability under the applied handling conditions was not evaluated. Future studies should include larger cohorts across a broader severity spectrum, document treatment and sampling times, incorporate systematic lactate measurements, and assess extraction recovery, dilutional linearity, interference and stability for each sample type before evaluating clinical utility.

## 5. Conclusions

This pilot study did not establish the usefulness of serum or faecal IL-1β, IL-6, and IL-8 for monitoring clinical status and postoperative recovery in horses with surgical colic. Faecal sampling offers a potentially non-invasive approach, but its application at admission was limited by the inability to obtain faecal material from some horses with colic. The nominally lower faecal estimates in colic horses cannot be interpreted as evidence of less intestinal inflammation without validation of the assay for equine faecal extracts. Further evaluation requires validated measurements and larger cohorts spanning a broader range of disease severity, with treatment effects and sampling times taken into account.

## Figures and Tables

**Table 1:** Horse identifiers (C followed by a number), age (years), clinical characteristics, postoperative course, and sample availability in horses with surgical colic.

Horse	Age	Sex	Lesion and Associated Findings	Surgical Procedure	Postoperative Course	Sampling Limitation
C2	3	Male	Right dorsal displacement of the colon; distended small intestine Associated observation: free peritoneal fluid	Decompression and repositioning	Diarrhoea and suspected laminitis	Rectal faecal sampling was not considered safe at D0
C7	15	Female	Torsion of colon and caecum with secondary small-intestinal torsion	Decompression and repositioning	Uneventful	None
C8	14	Female	180° colonic torsion; caecal adhesions; dilated small intestine	Decompression, repositioning, adhesion dissection and enterotomy	Transient deterioration at 24 h, followed by recovery	D0 faeces unavailable
C9	8	Male	180° colonic torsion with small- and large-intestinal gaseous distension	Decompression and repositioning	Uneventful	D0 faeces unavailable; D1 serum collected; the sample was damaged before analysis and could not be analysed
C10	7	Female	Retroflexio coli with small-intestinal distension	Decompression and repositioning	Uneventful	None
C12	6	Male	Mesenteric volvulus; thickened intestinal wall; sand in colon/faeces	Decompression and repositioning	Persistent abdominal pain; lidocaine CRI for 48 h	None
C13	11	Male	Non-strangulating herniation through the epiploic foramen; entrapment involving omentum and part of the jejunum; dilated small intestine	Caecal decompression, small-intestinal milking and foramen closure	Uneventful	None
C16	8	Female	Dilated small-intestinal loops and stomach	Small-intestinal milking, enterotomy, decompression and repositioning	Recovery with mild signs of pain	None

**Table 2:** Serum IL-8 concentrations (pg/mL), reported as median and interquartile range (Q1–Q3).

Group	Time	N	Median [Q1–Q3]
Healthy	D0	6	257.59 [239.09–263.37]
Colic	D0	8	220.63 [202.54–263.31]
Colic	D1	7	205.90 [172.16–251.77]
Colic	D7	8	214.75 [178.07–268.65]

**Table 3:** Faecal cytokine estimates (pg/mL of extract), reported as median and interquartile range (Q1–Q3). H, healthy controls; C, colic horses. All between-group comparisons are exploratory, and *p* values are interpreted as nominal.

Cytokine	Day	H N	H Median [Q1–Q3]	C N	C Median [Q1–Q3]	Between-Group
IL-1β	D0	6	9167.35 [5664.25–12,300.73]	5	4223.33 [3668.35–7259.54]	*p* > 0.05
IL-1β	D1	6	6991.40 [5942.34–9087.61]	8	5378.68 [4499.73–6362.81]	*p* > 0.05
IL-1β	D7	6	10,676.34 [9930.06–11,766.82]	8	4248.73 [3844.30–6689.17]	*p* = 0.033
IL-6	D0	6	172.47 [135.09–201.07]	5	112.81 [107.23–146.24]	*p* > 0.05
IL-6	D1	6	180.85 [140.66–270.24]	8	133.70 [100.82–165.75]	*p* = 0.045
IL-6	D7	6	157.38 [144.00–201.04]	8	157.96 [113.90–181.42]	*p* > 0.05
IL-8	D0	6	37.54 [29.73–48.35]	5	14.16 [9.62–26.30]	*p* > 0.05
IL-8	D1	6	46.45 [22.50–56.67]	8	24.40 [17.38–40.91]	*p* > 0.05
IL-8	D7	6	49.71 [36.33–97.50]	8	29.60 [12.70–33.15]	*p* = 0.004

## Data Availability

The data that support the findings of this study are available from the corresponding author upon reasonable request.

## References

[1] Tinker M.K., White N.A., Lessard P., Thatcher C.D., Pelzer K.D., Davis B., Carmel D.K. (1997). Prospective Study of Equine Colic Incidence and Mortality. Equine Vet. J..

[2] Fikri F., Hendrawan D., Wicaksono A.P., Purnomo A., Khairani S., Chhetri S., Purnama M.T.E., Çalışkan H. (2024). Colic Incidence, Risk Factors, and Therapeutic Management in a Working Horse Population in Tuban, Indonesia. Vet. World.

[3] Siwińska N., Żak-Bochenek A., Paszkowska M., Karczewski M., Długopolska D., Haider W. (2022). Retrospective Evaluation of the Most Frequently Observed Histological Changes in Duodenal and Rectal Mucosal Biopsies in Horses with Recurrent Colic. Animals.

[4] Moore J.N., Vandenplas M.L. (2014). Is It the Systemic Inflammatory Response Syndrome or Endotoxemia in Horses with Colic?. Vet. Clin. N. Am. Equine Pract..

[5] Hajimohammadi A., Ghane M., Ghari Tehrani M., Paravar B., Mirzaei A., Razavi S., Nikzad M. (2023). Association of the Severity of Colic in Horses with Oxidative Stress Biomarkers, Acute-Phase Proteins, and Certain Trace Elements. J. Equine Sci..

[6] Ludwig E.K., Hobbs K.J., McKinney-Aguirre C.A., Gonzalez L.M. (2023). Biomarkers of Intestinal Injury in Colic. Animals.

[7] Magri M. (2023). Colic Horse: A Diagnostic Challenge. Equine Vet. Educ..

[8] Cahill C.M., Rogers J.T. (2008). Interleukin (IL) 1β Induction of IL-6 Is Mediated by a Novel Phosphatidylinositol 3-Kinase-Dependent AKT/IκB Kinase α Pathway Targeting Activator Protein-1. J. Biol. Chem..

[9] Yadav S., Prasannan A., Venkatachalam K., Binesh A. (2025). Exploring the Mechanism and Crosstalk between IL-6 and IL- 1β on M2 Macrophages under Metabolic Stress Conditions. Cytokine.

[10] Kim G.-Y., Lee J.-W., Ryu H.-C., Wei J.-D., Seong C.-M., Kim J.-H. (2010). Proinflammatory Cytokine IL-1β Stimulates IL-8 Synthesis in Mast Cells via a Leukotriene B4 Receptor 2-Linked Pathway, Contributing to Angiogenesis. J. Immunol..

[11] Eder C. (2009). Mechanisms of Interleukin-1β Release. Immunobiology.

[12] Kaneko N., Kurata M., Yamamoto T., Morikawa S., Masumoto J. (2019). The Role of Interleukin-1 in General Pathology. Inflamm. Regen..

[13] Jawa R.S., Anillo S., Huntoon K., Baumann H., Kulaylat M. (2011). Analytic Review: Interleukin-6 in Surgery, Trauma, and Critical Care: Part I: Basic Science. J. Intensive Care Med..

[14] Epstein A., Nir E., Eyngor M., Eldar A., Bdolah-Abram T., Kelmer E., Steinman A., Bruchim Y. (2016). Dynamic of Cytokine Gene Transcription (TNF-α, IL-1β, IL-6, IL-8) in Surgically Treated Colic Horses by Use of Real-Time PCR (RT-PCR). Isr. J. Vet. Med..

[15] Barton M.H., Collatos C. (1999). Tumor Necrosis Factor and Interleukin-6 Activity and Endotoxin Concentration in Peritoneal Fluid and Blood of Horses with Acute Abdominal Disease. J. Vet. Intern. Med..

[16] Teschner D., Rieger M., Koopmann C., Gehlen H. (2015). Procalcitonin in horses with an acute colic. Pferdeheilkunde Equine Med..

[17] El Sayed Zaki M., Alsayed M.A.L., Shrief R. (2020). Study of the Diagnostic Value of Interleukin-6 and Interleukin-8 in Children with Acute Gastroenteritis. Germs.

[18] Saiki T., Mitsuyama K., Toyonga A. (1998). Detection of Pro- and Anti-Inflammatory Cytokines in Stools of Patients with Inflammatory Bowel Disease. Scand. J. Gastroenterol..

[19] Di Iorio A., Ferrucci L., Sparvieri E., Cherubini A., Volpato S., Corsi A., Bonafè M., Franceschi C., Abate G., Paganelli R. (2003). Serum IL-1β Levels in Health and Disease: A Population-Based Study. ‘The InCHIANTI Study’. Cytokine.

[20] Opal S.M., DePalo V.A. (2000). Anti-Inflammatory Cytokines. Chest.

[21] Koch A.E., Polverini P.J., Kunkel S.L., Harlow L.A., DiPietro L.A., Elner V.M., Elner S.G., Strieter R.M. (1992). Interleukin-8 as a Macrophage-Derived Mediator of Angiogenesis. Science.

[22] Burton A.B., Wagner B., Erb H.N., Ainsworth D.M. (2009). Serum Interleukin-6 (IL-6) and IL-10 Concentrations in Normal and Septic Neonatal Foals. Vet. Immunol. Immunopathol..

[23] Basinska K., Marycz K., Śmieszek A., Nicpoń J. (2015). The Production and Distribution of IL-6 and TNF-α in Subcutaneous Adipose Tissue and Their Correlation with Serum Concentrations in Welsh Ponies with Equine Metabolic Syndrome. J. Vet. Sci..

[24] Chen J.-W., Stefanovski D., Haughan J., Jiang Z., Soma L.R., Robinson M.A. (2025). Positive Correlation between Interleukin (IL) 1 Beta to IL-1 Receptor Antagonist Levels in Standardbred Racehorses Prior to Racing. Vet. Immunol. Immunopathol..

[25] Witkowska-Piłaszewicz O., Pingwara R., Winnicka A. (2020). The Effect of Physical Training on Peripheral Blood Mononuclear Cell Ex Vivo Proliferation, Differentiation, Activity, and Reactive Oxygen Species Production in Racehorses. Antioxidants.

[26] Rettig T.C.D., Verwijmeren L., Dijkstra I.M., Boerma D., Van De Garde E.M.W., Noordzij P.G. (2016). Postoperative Interleukin-6 Level and Early Detection of Complications After Elective Major Abdominal Surgery. Ann. Surg..

[27] Nowicka B., Polkowska I., Zeliszewska-Duk P., Torres A., Duk M. (2025). Molecular Assessment of Plasma Concentrations of Selected Adipokines and IL-8 in Horses with Back Pain and Comorbid Asthma—Based on Clinical Cases. Animals.

[28] Nieto J.E., Macdonald M.H., Braim A.E.P., Aleman M. (2009). Effect of Lipopolysaccharide Infusion on Gene Expression of Inflammatory Cytokines in Normal Horses in Vivo. Equine Vet. J..

[29] Muroi C., Hugelshofer M., Seule M., Keller E. (2014). The Impact of Nonsteroidal Anti-Inflammatory Drugs on Inflammatory Response After Aneurysmal Subarachnoid Hemorrhage. Neurocrit. Care.

[30] Yan Y., Guo T.-M., Zhu C. (2018). Effects of Nonsteroidal Anti-Inflammatory Drugs on Serum Proinflammatory Cytokines in the Treatment of Ankylosing Spondylitis. Biochem. Cell Biol..

[31] Kongwut S., Soontornvipart K., Sarikaphuti M., Makoom P., Nganvongpanit K. (2015). Effect of Serum IL-1beta of PCSO-524 and Firocoxib in Dogs Undergoing Medial Patellar Luxation Repair. Thai J. Vet. Med..

[32] Brown K.A., Gregorio E.N., Barot D., Usimaki A., Linardi R.L., Missanelli J.R., You Y., Robinson M.A., Ortved K.F. (2024). Single-Dose Nonsteroidal Anti-Inflammatory Drugs in Horses Have No Impact on Concentrations of Cytokines or Growth Factors in Autologous Protein Solution and Platelet-Rich Plasma. Am. J. Vet. Res..

[33] Republic of Poland Act of 15 January 2015 on the Protection of Animals Used for Scientific or Educational Purposes. Journal of Laws 2015, Item 266. https://eli.gov.pl/eli/DU/2015/266/ogl.

[34] Republic of Poland Act of 17 November 2021 Amending the Act on the Protection of Animals Used for Scientific or Educational Purposes. Journal of Laws 2021, Item 2338. https://eli.gov.pl/eli/DU/2021/2338/ogl.

